# Full-length transcriptome profiling for fruit development in *Diospyros oleifera* using nanopore sequencing

**DOI:** 10.1186/s12863-023-01105-w

**Published:** 2023-03-13

**Authors:** Yang Xu, Cui-yu Liu, Wen-qiang Cheng, Kai-yun Wu, Bang-chu Gong

**Affiliations:** Research Institute of Subtropical Forestry, Chinese Academy of Forestry, No. 76, Daqiao Road, Fuyang District, Hangzhou, China

**Keywords:** *Diospyros oleifera*, Fruit development, Nanopore sequencing, Full-length transcriptome

## Abstract

**Objectives:**

*Diospyros oleifera*, one of the most economically important *Diospyros species*, is an ideal model for studying the fruit development of persimmon. While, the lack of whole-transcriptome has hindered the complex transcriptional regulation mechanisms of sugar and tannin during fruit development.

**Data description:**

We applied Oxford Nanopore Technologies to six developmental stage of fruit from *D. oleifera* for use in transcriptome sequencing. As a result of full-length transcriptome sequencing, 55.87 Gb of clean data were generated. After mapping onto the reference genome of *D. oleifera*, 51,588 full-length collapsing transcripts, including 2,727 new gene loci and 43,223 transcripts, were obtained. Comprehensively annotated, 38,086 of new transcripts were functional annotation, and 972 lncRNAs, 7,159 AS events were predicted. Here, we released the transcriptome database of *D. oleifera* at different stage of fruit development,which will provide a fundamention of to investigatethe transcript structure, variants and evolution of persimmon.

## Objective

There are approximately 500 species in the genus *Diospyros*, which range in ploidy level from diploid (2n = 2x = 30) to nonaploid (2n = 9x = 135) [1, 2]. Among these species, *Diospyros oleifera* and *Diospyros kaki* have been cultivated as important fruit crops in east Asia for centuries., these edible fruitstare rich in vitamins, sugars, nutrients, and antioxidants that are important for optimum health [3, 4]. Furthermore, *D. oleifera* is diploid (2n = 2 ×  = 30) and is closely related to *D. kaki* (2n = 6 ×  = 90) [4, 5]. As an added advantage, *D. oleifera* could be used as a model plant for studies of *Diospyros* [4, 6, 7].

Fruit development plays an important role in the life cycle of higher plants. *D.oleifera* will also be a potential model plant for studies of sugar synthesis and transformation, tannin formation and deastringency, coordination network of tannin and sugar during fruit development. Although we have reported the *D. oleifera* genome [6], transcript profile data on *Diospyros* during fruit development is insufficient compared with those of other fruit [8–10]. Even, no full-length transcriptome of *D.oleifera* has been reported. In this study, the ONT was used to generate large-scale full-length transcripts and collect the gene expression profile of *D. oleifera* fruit development*.*These data will provide gene sequence information and comprehensive understanding of the fruit development of persimmon.

## Data description

The fruit flesh of *D. oleifera* were obtained from 10 years-old plant in LanXi Plant Nursery (E, 119°28′27.274″; N, 29°8′48.946″), which located in LanXi City, Zhejiang Province. Three biological replicates were harvested at six development stages: (10 days after pollination (DAP)(T01-T03), 40 DAP(T04-T06), 100 DAP(T07-T09), 160 DAP(T10-T12), 180 DAP(T13-T15) and 200 DAP(T16-T18)). An RNeasy Plant Mini kit (Qiangen, 74,904) was used to extract total RNA, which was then treated with RNase-free DNase I (TAKARA, D2215). Nanodrop 2000 and Agilent 2100 were used to assess RNA quality (Data file 1). 1ug of total RNA was used for cDNA libraries with the protocol of Oxford Nanopore Technologies (ONT)(Oxford Nanopore Technologies, Oxford, UK). FLO-MIN109 flowcells were used to run the final cDNA libraries at Biomarker Technology Company (Beijing, Chinai), using the PromethION platform.

First, raw reads were filtered under the standard of an average read quality score is not lower than 7 and a read length is not lower than 500 bases [11]. Ribosomal RNA (rRNA) were discarded after mapping to rRNA database. Full-length transcripts (FLs) were identifiedusing the primers at both ends of cleaned reads. Full-length andnon-chemiric (FLNC) transcripts were clustered via mapping to *D. oleifera* reference genome [6] with mimimap2 [12]. Then consensus isoforms were obtained from each cluster using pinfish. Mapped reads were further collapsed to remove redundant FLs with 85% of min-coverage and 90% of min-identity by cDNA_Cupcake package. 5’ difference was not considered when collapsing redundant transcripts. A single transcript of fusion candidates must conform the following criteria: (1) map loci must be more than or equal to 2, (2) coverage for each loci is >  = 5% and minimum coverage in bp is more than or equal to 1 bp, (3) total coverage is >  = 95%, (4) distance between the loci is not shorter than 10 kb.

Alternative splicing (AS) events and alternative polyadenylation (APA) events were identified by AStalavista tool (v3.2) [13] and TAPIS [14], respectively. The coding sequences and corresponding amino acid sequences was predicted by TransDecoder v3.0.0 [15]. GMAP (http://research-pub.gene.com/gmap/, v2017-11–15) was used to identify new transcripts. Four computational approaches include Coding Potential Calculator (CPC) [16], Coding-Non-Coding Index (CNCI) [17], Coding Potential Assessment Tool (CPAT) [18], and Pfam reference protein databases [19] were combined to sort non-protein cosubsequent to filtering. Long Non-coding RNAs (lncRNAs) were identified under the standard of at least 200 nt and two exons. Target genes regulating by identified lncRNAs were predicted using LncTar (v1.0) [20].

The annotations of transcripts were performed with e-values of 1e^−5^ on eight databases, including non-redundant protein sequence database(NR) [21], the database of Homologous protein family (Pfam) [19], eukaryotic Ortholog Groups(KOG) [22], Clusters of Orthologous Groups of proteins [23], evolutionary genealogy of genes: Non-supervised Orthologous Groups(eggNOG) [24], a manually annotated, non-redundant protein sequence database(Swiss-Prot) [25], Kyoto Encyclopedia of Genes and Genomes (KEGG) [26] and Gene Ontology(GO) [27].

Full-length reads were mapped to the reference transcriptome sequence, and then reads with match quality above 5 after mapping were further used to quantify. The absolute CPM (counts per million) value more than 0.1 was considered as a reliable expression. Differential expression analysis of two samples was performed using the DESeq R package (1.18.0) [28] with the following criteria: FDR < 0.01 and fold-change ≥ 2.

We applied Oxford Nanopore Technologies on six developmental stages of *D. oleifera* fruits for transcriptome sequencing (Data file 1). As a result, a total of 55.87 Gb clean data were generated (Data file 2, Data set 1- Data set 18). After mapping onto the reference genome of *D. oleifera* and discarding rRNA*,* we obtained 1,190,459 to 3,046,317 full-length reads (FL reads) from each sample (Data file 3, Data set 1-Data set 18). Though clustering, we obtained 51,588 full-length collapsing transcripts with an average length of 1,311 bp. And then, 43,223 new transcripts were identified among these collapsing redundant transcripts. Comprehensively annotated, 38,086 of new transcripts were functional annotation. In total, 35, 243 genes were detected, including 32,406 genes with functional annotation and 2,727 newly identified genes (Data file 4). 7,159 Alternative Splicing (AS) events were detected, as shown in Data file 5 and Data file 6 including 100 mutually exclusive exons, 2,115 intron retention (IR) events, 1,698 exon skipping (ES) events, 1,553 5' AS (Alt. 5') sites and 1,693 3'AS (Alt. 3') sites. We further detected 9274–13,034 APA events (Data file 7) and 14 -52 fusion genes (Data file 8) in each sample. 972 lncRNAs were screened and classified as shown in Data file 9. And, Data file 10 shows the target genes for 933 lncRNAs. We also found that 19, 276 genes and 39,969 transcripts were diferentially expressed during fruit development. Moreover, differentially expressed genes (DEGs) and differentially expressed transcripts (DETs) between all pairs of adjacent stages were also shown in Data file 11 and Data file 12. The dataset not only can offer fundamental genetic information to investigate transcript structure, variants and evolution of persimmon, but also can offer a reference to further analyse the transcriptome in persimmon fruit development (Table 1).

**Table 1:** Overview of data files/data sets

Label	Name of data file/data set	File types (file extension)	Data repository and identifier (DOI or accession number)
Data file 1	Summary of sequencing sample and strategies in this study	MS Word fle(.docx)	figshare(https://doi.org/10.6084/m9.figshare.19314470) [29]
Data file 2	Statistic of ONT-sequencing in this study	MS Word fle(.docx)	figshare(https://doi.org/10.6084/m9.figshare.19314515) [30]
Data file 3	Read number and length distribution of FLNC and Collapse transcripts after ONT-Seq analysis	MS Word fle(.docx)	figshare(https://doi.org/10.6084/m9.figshare.19314524) [31]
Data file 4	Gene information and database annotations	MS Excel file(.xls)	figshare(https://doi.org/10.6084/m9.figshare.19314536) [32]
Data file 5	The total number of AS events in detected genes and transcripts	MS Word fle(.docx)	figshare(https://doi.org/10.6084/m9.figshare.19314539) [33]
Data file 6	The characteristics of AS events in each sample	MS Excel file(.xls)	figshare(https://doi.org/10.6084/m9.figshare.19314545) [34]
Data file 7	The statistical lists of APA events for each sample	MS Excel file(.xls)	figshare(https://doi.org/10.6084/m9.figshare.19314548) [35]
Data file 8	The statistical list of all fusion gene for each sample	MS Excel file(.xls)	figshare(https://doi.org/10.6084/m9.figshare.19314563) [36]
Data file 9	The result of LncRNAs classifications	Portable Document Format file (.pdf)	figshare(https://doi.org/10.6084/m9.figshare.19314566) [37]
Data file 10	The information of target genes of these 933 lncRNAs	MS Excel file(.xls)	figshare(https://doi.org/10.6084/m9.figshare.19314569) [38]
Data file 11	The quantitative gene expression of all DEGs	MS Excel file(.xls)	figshare(https://doi.org/10.6084/m9.figshare.19314572) [39]
Data file 12	The quantitative gene expression of all DETs	MS Excel file(.xls)	figshare(https://doi.org/10.6084/m9.figshare.19314584) [40]
Data set 1	Nanopore sequencing reads of T01	fastq (.fastq.gz)	NCBI Sequence Read Archive(https://identifiers.org/ncbi/insdc.sra:SRR14918124) [41]
Data set 2	Nanopore sequencing reads of T02	fastq (.fastq.gz)	NCBI Sequence Read Archive(https://identifiers.org/ncbi/insdc.sra:SRR14918123) [42]
Data set 3	Nanopore sequencing reads of T03	fastq (.fastq.gz)	NCBI Sequence Read Archive(https://identifiers.org/ncbi/insdc.sra:SRR14918114) [43]
Data set 4	Nanopore sequencing reads of T04	fastq (.fastq.gz)	NCBI Sequence Read Archive(https://identifiers.org/ncbi/insdc.sra:SRR14918113) [44]
Data set 5	Nanopore sequencing reads of T05	fastq (.fastq.gz)	NCBI Sequence Read Archive(https://identifiers.org/ncbi/insdc.sra:SRR14918112) [45]
Data set 6	Nanopore sequencing reads of T06	fastq (.fastq.gz)	NCBI Sequence Read Archive(https://identifiers.org/ncbi/insdc.sra:SRR14918111) [46]
Data set 7	Nanopore sequencing reads of T07	fastq (.fastq.gz)	NCBI Sequence Read Archive(https://identifiers.org/ncbi/insdc.sra:SRR14918110) [47]
Data set 8	Nanopore sequencing reads of T08	fastq (.fastq.gz)	NCBI Sequence Read Archive(https://identifiers.org/ncbi/insdc.sra:SRR14918109) [48]
Data set 9	Nanopore sequencing reads of T09	fastq (.fastq.gz)	NCBI Sequence Read Archive(https://identifiers.org/ncbi/insdc.sra:SRR14918108) [49]
Data set 10	Nanopore sequencing reads of T10	fastq (.fastq.gz)	NCBI Sequence Read Archive(https://identifiers.org/ncbi/insdc.sra:SRR14918107) [50]
Data set 11	Nanopore sequencing reads of T11	fastq (.fastq.gz)	NCBI Sequence Read Archive(https://identifiers.org/ncbi/insdc.sra:SRR14918122) [51]
Data set 12	Nanopore sequencing reads of T12	fastq (.fastq.gz)	NCBI Sequence Read Archive(https://identifiers.org/ncbi/insdc.sra:SRR14918121) [52]
Data set 13	Nanopore sequencing reads of T13	fastq (.fastq.gz)	NCBI Sequence Read Archive(https://identifiers.org/ncbi/insdc.sra:SRR14918120) [53]
Data set 14	Nanopore sequencing reads of T14	fastq (.fastq.gz)	NCBI Sequence Read Archive(https://identifiers.org/ncbi/insdc.sra:SRR14918119) [54]
Data set 15	Nanopore sequencing reads of T15	fastq (.fastq.gz)	NCBI Sequence Read Archive(https://identifiers.org/ncbi/insdc.sra:SRR14918118) [55]
Data set 16	Nanopore sequencing reads of T16	fastq (.fastq.gz)	NCBI Sequence Read Archive(https://identifiers.org/ncbi/insdc.sra:SRR14918117) [56]
Data set 17	Nanopore sequencing reads of T17	fastq (.fastq.gz)	NCBI Sequence Read Archive(https://identifiers.org/ncbi/insdc.sra:SRR14918116) [57]
Data set 18	Nanopore sequencing reads of T18	fastq (.fastq.gz)	NCBI Sequence Read Archive(https://identifiers.org/ncbi/insdc.sra:SRR14918115) [58]

## Limitations

Here, we describe the transcriptomic profile of *D. oleifera* during different stages of fruit development. One limitation of our study is that qRT-PCR analysis should be conducted to validate the identified patterns of differential gene expression here. Long-read sequencing platforms have the capability to sequence entire cDNA molecules end-to-end, while, the accuracy of these long reads is usual lower than Illumina sequencing. So, high-accuracy and short reads obtained from Illumina sequencing should supply to offset the reduced accuracy of these long reads from nanopore sequencing.

## Data Availability

Data described in this Data note can be freely and openly accessed on NCBI under Bioproject ID PRJNA736836, accession number SRR14918107-SRR14918124. Please see The details were showed in Table 1 and references [29–58].

## Abbreviations

DAP: Days after pollination
ONT: Oxford Nanopore Technologies
rRNA: Ribosomal RNA
FLs: Full-length transcripts
FLNC: Full-length, non-chemiric
AS: Alternative splicing
APA: Alternative polyadenylation
CPC: Coding Potential Calculator
CNCI: Coding-Non-Coding Index
CPAT: Coding Potential Assessment Tool
NR: Non-redundant protein sequence database
Pfam: The database of Homologous protein family
KOG: Eukaryotic Ortholog Groups
COG: Clusters of Orthologous Groups of proteins
eggNOG: Evolutionary genealogy of genes: Non-supervised Orthologous Groups
Swiss-Prot: A manually annotated, non-redundant protein sequence database
KEGG: Kyoto Encyclopedia of Genes and Genomes
GO: Gene Ontology
lncRNAs: Non-coding RNAs
CPM: Counts per million
FL reads: Full-length reads
IR: Intron retention
ES: Exon skipping
DEGs: Differentially expressed genes
DETs: Differentially expressed transcripts
